# STAT3 Regulates Proliferation and Survival of CD8^+^ T Cells: Enhances Effector Responses to HSV-1 Infection, and Inhibits IL-10^+^ Regulatory CD8^+^ T Cells in Autoimmune Uveitis

**DOI:** 10.1155/2013/359674

**Published:** 2013-09-24

**Authors:** Cheng-Rong Yu, Ivy M. Dambuza, Yong-Jun Lee, Gregory M. Frank, Charles E. Egwuagu

**Affiliations:** ^1^Molecular Immunology Section, National Eye Institute, National Institutes of Allergy and Viral Diseases, National Institutes of Health, Building 10, Room 10N109A, 10 Center Drive, Bethesda, MD 20892-1857, USA; ^2^Cellular Biology and Viral Immunology Section, National Institutes of Allergy and Viral Diseases, National Institutes of Health, Building 33, Room 2E13, 33 North Drive, Bethesda, MD, USA

## Abstract

STAT3 regulates CD4^+^ T cell survival and differentiation. However, its effects on CD8^+^ T cells are not well understood. Here, we show that in comparison to WT CD8^+^ T cells, STAT3-deficient CD8^+^ T cells exhibit a preactivated memory-like phenotype, produce more IL-2, proliferate faster, and are more sensitive to activation-induced cell death (AICD). The enhanced proliferation and sensitivity to AICD correlated with downregulation of class-O forkhead transcription factors (FoxO1, FoxO3A), p21^waf1^, p27^KIP1^, Bcl-2, OX-40, and upregulation of FasL, Bax, and Bad. We examined whether STAT3-deficient CD8^+^ T cells can mount effective response during herpes simplex virus (HSV-1) infection and experimental autoimmune uveitis (EAU). Compared to WT mice, HSV-1-infected STAT3-deficient mice (STAT3KO) produced less IFN-*γ* and virus-specific KLRG-1^+^ CD8^+^ T cells. STAT3KO mice are also resistant to EAU and produced less IL-17-producing Tc17 cells. Resistance of STAT3KO to EAU correlated with marked expansion of IL-10-producing regulatory CD8^+^ T cells (CD8-Treg) implicated in recovery from autoimmune encephalomyelitis. Thus, increases of IL-6-induced STAT3 activation observed during inflammation may inhibit expansion of CD8-Tregs, thereby impeding recovery from uveitis. These results suggest that STAT3 is a potential therapeutic target for upregulating CD8^+^ T cell-mediated responses to viruses and suggest the successful therapeutic targeting of STAT3 as treatment for uveitis, derived, in part, from promoting CD8-Treg expansion.

## 1. Introduction

STAT3 was originally described as an acute-phase response factor (APRF) induced by IL-6 [1, 2], and many cytokines produced by innate and adaptive immune cells including IL-10, IL-21, IL-23, and IL-27 have now been shown to induce STAT3 activation [3]. Understanding the myriad of functions attributed to STAT3 in host immune responses was limited by the fact that STAT3 deletion is embryonically lethal [4]. To circumvent this limitation, mice with targeted deletion of STAT3 in specific cell types have been generated by use of the Cre-loxP recombination technology [4–8]. Mice with deletion of STAT3 in T cells generated by mating STAT3^fl/fl^ and LCK-Cre mice suggested that STAT3 mediates IL-6-dependent T cell proliferation by preventing apoptosis [9]. Subsequent studies using mice with targeted deletion of STAT3 in the CD4 compartment using CD4-Cre mice revealed that STAT3 inhibits IL-2 production and CD4^+^ T cell proliferation by upregulating the expression of class-O forkhead transcription factors (Fox O) and promoting the sequestration of NF-*κ*B in the cytoplasm [10]. However, it is not known whether STAT3 also regulates proliferation, effector functions, and survival of CD8^+^ T cells. 

In this study, we took advantage of the fact that the CD4 promoter is activated at the CD4/CD8 double positive stage of T cell development, and by mating the CD4-Cre and STAT3^f/f^ mouse strains, we generated mice with deletion of STAT3 in CD8, as well as, the CD4 T cell compartment. We describe here studies characterizing the phenotype of STAT3-deficient CD8^+^ T cell (CD8-STAT3KO) and the role STAT3 plays in controlling CD8^+^ T cell proliferation, IL-2 production, and activation-induced cell death (AICD) *in vitro*. We also examined the effect of STAT3 in CD8^+^ T cell functions *in vivo*, specifically in host immunity to HSV-1 infection and autoimmune uveitis. 

## 2. Materials and Methods

### 2.1. Animals and Reagents

Six-to-8-week old C57BL/6 mice were purchased from Jackson Laboratory (Bar Harbor, ME). CD4-CRE transgenic mice were purchased from Taconic (Taconic, Hudson, NY). Dr. David Levy (New York University, New York, NY) provided the STAT3^fl/fl^ mice. Mice with loss of STAT3 in CD4 compartment (STAT3KO mice) were generated by breeding CD4-CRE and Stat3^fl/fl^ mouse strains and had previously been described [7]. Animal care and use were in compliance with NIH guidelines. 

### 2.2. Herpes Simplex Virus 1 (HSV-1) Infection

Recombinant HSV-1 (HSV-1) virus, RE-pICP0-EGFP [11], was kindly provided by Paul R. Kinchington (University of Pittsburgh, School of Medicine, Pittsburgh, Pennsylvania). HSV-1 was grown in Vero cells. The intact virions were purified on OptiPrep gradients according to the manufacturer's instructions (Accurate Chemical & Scientific Corp., Westbury, NY) and quantified as plaque forming units/mL (PFU/mL) on Vero cell monolayer [11]. STAT3^f/f^ (WT) and CD4-STAT3KO mice (12 mice per group) were infected with 1.0 × 10^6^ PFU/mL. Infectivity was established by detection of tetramer positive HSV-1-specific CD8 T cells responsive to HSV-1 gB tetramers. HSV-1 gB 498–505 peptide was synthesized and HPLC-purified by Invitrogen. H-2K^b^ HSV-1 gB (SSIEFARL/PE) tetramers were synthesized by NIH tetramer facility, Emory University, Atlanta. GA. Dimer PE-H-2Kb: Ig Fusion protein was purchased from BD Bioscience, and gB peptide was loaded as recommended by the manufacturer's instructions.

### 2.3. Induction of Experimental Autoimmune Uveitis (EAU)

Age matched STAT3^f/f^ mice or STAT3KO mice on a C57BL/6J background were subcutaneously injected with 150 *μ*g of bovine interphotoreceptor retinoid-binding protein (IRBP) and 300 *μ*g of human IRBP peptide (amino acid residues 1–20) per mouse in 0.2 mL emulsion (1 : 1 v/v with CFA containing *Mycobacterium tuberculosis* strain H37RA (2.5 mg/mL). Mice also received *Bordetella pertussis* toxin (0.3 *μ*g/mouse) via intraperitoneal injection. The eyes were fixed in 10% buffered formalin and serially sectioned in the vertical pupillary-optic nerve plane. All sections were stained with H&E. Disease severity was scored as described previously [12].

### 2.4. CD8^+^ T cell Isolation and Cultures

CD8^+^ and CD4^+^ T cells were isolated from lymph nodes (LN) and spleens of 6–8-week-old WT and STAT3KO mice. CD3 T cells were enriched using CD3-cell-enrichment column by negative selection. Further purification of CD8^+^ or CD4^+^ T cells was performed using anti-CD4 or -CD8-Ab conjugated magnetic beads (Miltenyi). In some experiments, naïve CD8^+^ T cells with CD62L^Hi^ and CD44^Lo^ were sorted electronically on a FACSAria II sorter (BD bioscience, CA). For the generation of Tc0 cells, naïve CD8^+^ T cells were cultured in plate-bound anti-CD3 (10 *μ*g/mL) and soluble anti-CD28 Abs (3 *μ*g/mL) (Bio X-cell, West Lebanon, NH). For the Tc1 cells, naïve CD8^+^ T cells were cultured in plate-bound anti-CD3 (10 *μ*g/mL), and soluble anti-CD28 Abs (3 *μ*g/mL), IL-12 (10 ng/mL), and anti-IL-4 Abs (0 *μ*g/mL) as previously described [13].

### 2.5. FACS Analysis and Intracellular Cytokine Staining

Freshly isolated thymocytes, peripheral blood mononuclear cells (PBMC), LN, or splenocytes were used directly for FACS analysis. After washing in cold 1xPBS, the cells were stained with antibodies against CD3, CD4, CD8, CD44, CD62L (BD BioScience), or KLRG-1 (e-BioScience). Intracellular staining assay for detection of cytokines or proteins was performed using an intracellular cytokine staining Kit (BD Bioscience) as described [13]. The cells were stimulated with PMA and ionomycin for 4 hours with addition of brefeldin A (5 *μ*g/mL) after 2 hours of stimulation. FACS analysis was performed on a Becton-Dickinson FACSCalibur (BD Pharmingen, San Diego, CA).

### 2.6. Lymphocyte Proliferation and Apoptosis Assays

Purified naïve CD8^+^ T cells were cultured for 4 days under Tc0 or Tc1 polarizing condition. After 36 h, cultures were pulsed with [^3^H]-thymidine (0.5 *μ*Ci/10 *μ*L/well) for 12 additional hours and analyzed as described [14]. For CFSE dilution assay, cells were labeled as recommended by the manufacturer (Molecular Probe Inc., Eugene, OR) and as previously described [13]. Annexin-V and 7-AAD apoptosis staining kits were purchased from BD Bioscience, and assay was performed as recommended by manufacturer. FACS analysis was performed on a Becton-Dickinson FACSCalibur (BD Harlingen, San Diego, CA).

### 2.7. Quantitative and Semiquantitative RT PCR Analyses

Total RNA was extracted using the TRIzol reagent according to the procedures recommended by the manufacturer (Life Technologies, Gaithersburg, MD). All RNA samples were digested with RNase-free DNase 1 (Life Technologies) for 30 min, purified by phenol/chloroform extractions, and precipitated in 0.4 M LiCl. RNA integrity was verified by analysis of 18S and 28S ribosomal RNA expression on RNA gels. RNA (10 *μ*g), SuperScript III Reverse Transcriptase (Life Technologies, Gaithersburg, MD), and oligo (dT)_12−16_ were used for first-strand synthesis as previously described [15]. Samples were subjected to hot-start RT-PCR with gene-specific primers and AmpliTaq Gold DNA polymerase (Applied Biosystems, CA). Primers used for RT-PCR amplification are as follows. Amplification was conducted for 25 to 33 cycles of 30 s each at 95°C, 60°C, and 72°C, followed by a final 10 min extension at 72°C. First-strand synthesis containing each mRNA sample but no reverse transcriptase was performed to control for possible DNA contamination of mRNAs used as target for PCR amplification; failure to obtain RT-PCR products with any of the PCR amplimers confirmed the absence of contaminating DNA templates. PCR-amplified fragments were fractionated on agarose gels. All cDNA preparations used were suitable substrates for PCR amplification on the basis of efficient amplification of a *β*-actin sequence. Real-time PCR was performed on an ABI 7500 (Applied Biosystems), and PCR parameters were as recommended for the TaqMan Universal PCR kit Applied Biosystems. Primers and probes were purchased from (Applied Biosystems).

### 2.8. Cytokine Analysis

Multiplex ELISA of supernatants for cytokine secretion was performed using a commercial ELISA kit R&D Systems, Minneapolis, MN.

### 2.9. Statistical Analyses

Results of statistical analyses were expressed as mean ± SD and were analyzed by Student's *t*-test (*P < 0.05, **P < 0.01, ****P < 0.0001, and NS denotes not significant).

## 3. Results

### 3.1. STAT3-Deficient CD8^+^ T Cells Exhibit Activation Phenotype

**Figure 1:**
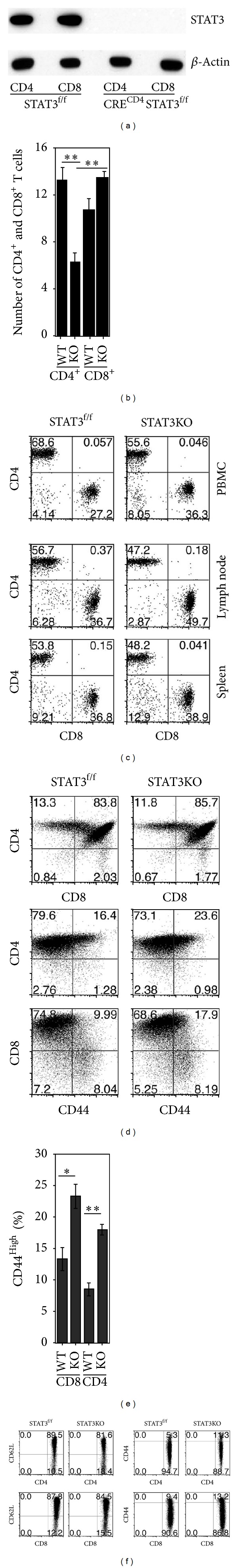
STAT3-deficient CD8^+^ T cells exhibit a preactivated phenotype. (a) Purified CD4^+^ or CD8^+^ T cells derived from the spleen and LN of control (STAT3^f/f^) and CD4-STAT3KO mice (CRE^CD4^STAT3^f/f^) were analyzed by western blotting. (b) Eight-week-old WT or STAT3KO mice were euthanized, and lymph nodes (LN) and spleen were harvested. Total CD4^+^ and CD8^+^ lymphocytes isolated from LN and spleen were quantified on a Vi-CELL Cell Viability Analyzer (Beckman Coulter). (c) Freshly isolated T cells from the PBMC, LN, or spleen were stained with Abs to CD3, CD4, or CD8 and analyzed by FACS. (d, e, f) Relative amounts of activated T cells in unstimulated thymocytes (d, e) or spleen (f) of the WT or STAT3KO mice were determined by FACS analysis of CD44^+^ or CD62L^+^ T cells. Plots (d, e, f) were gated on CD3, and numbers in quadrants represent percentage of T cells expressing CD4, CD8, or CD44. Data represent at least 3 independent experiments.

The CD8^+^ T cell plays a central role in host immunity against viruses and other intracellular pathogens. Following pathogen recognition in context of MHC class I on antigen presenting cells (APCs), the naïve CD8^+^ T cell differentiates into Tc1, Tc2, or Tc17 cells and begins expressing high levels of KRLG-1 (killer lectin-like receptor subfamily G member 1) and the proinflammatory cytokine, IFN-*γ* that mediate their biological activities [16–19]. In this study, we analyzed CD4-STAT3KO mice with targeted deletion of *Stat3* in the CD4 compartment to investigate the potential involvement of STAT3 pathway in CD8^+^ T cell development and effector functions. Since the functional CD4 promoter is active at the CD4^+^CD8^+^ double positive stage of T cell development [20, 21], we expected that high expression of the Cre protein under the direction of a CD4 promoter element would lead to deletion of the STAT3 protein in both CD4^+^ and CD8^+^ T cells. To confirm that STAT3 is indeed deleted in CD4-STAT3KO T cells, we isolated CD4^+^ and CD8^+^ T cells from WT and CD4-STAT3KO mice, purified the cells by cell sorting, and prepared whole cell protein extracts. Western blot analysis of whole cell extracts prepared from sorted CD8^+^ or CD4^+^ T cells revealed complete deletion of STAT3 in both CD4^+^ and CD8^+^ T cells (Figure 1(a)). We then isolated CD3^+^ T cells from the blood, lymph nodes (LN), and spleen of the WT and CD4-STAT3KO mice and investigated whether the loss of STAT3 has disproportionate impact on CD4^+^ or CD8^+^ T cells. Analysis of the CD4^+^ T cell population showed a significant decrease in the number of CD4^+^ T cells in the CD4-STAT3KO compared to WT control (Figure 1(b)). The marked decrease in the number of resting and unstimulating CD4^+^ T cells in the CD4-STAT3KO mice is consistent with the role of STAT3 inducing expression of FoxO1 and FoxO3a, two class O forkhead transcription factors that contribute to maintenance of CD4^+^ T cells in resting or quiescence state [14]. Interestingly, we observed a significant increase in CD8^+^ T cells in the CD4-STAT3KO compared to WT mice (Figure 1(b)), suggesting that STAT3 may serve to maintain CD8^+^ T cells at low levels under noninflammatory condition. Consistent with the differential effects of STAT3 on resting CD4^+^ and CD8^+^ T cells, we observed an increase of CD8 : CD4 ratio in STAT3KO compared to WT counterparts (Figures 1(b) and 1(c)). In line with previous reports [14, 22], the STAT3-deficient CD8^+^ T cells exhibited an activation phenotype as indicated by the elevation of CD44 expression (Figures 1(d) and 1(e)) and reduced CD62L (Figure 1(f)).

### 3.2. STAT3 Suppresses Proliferation and Protects CD8-STAT3KO from IL-2-Induced AICD

**Figure 2:**
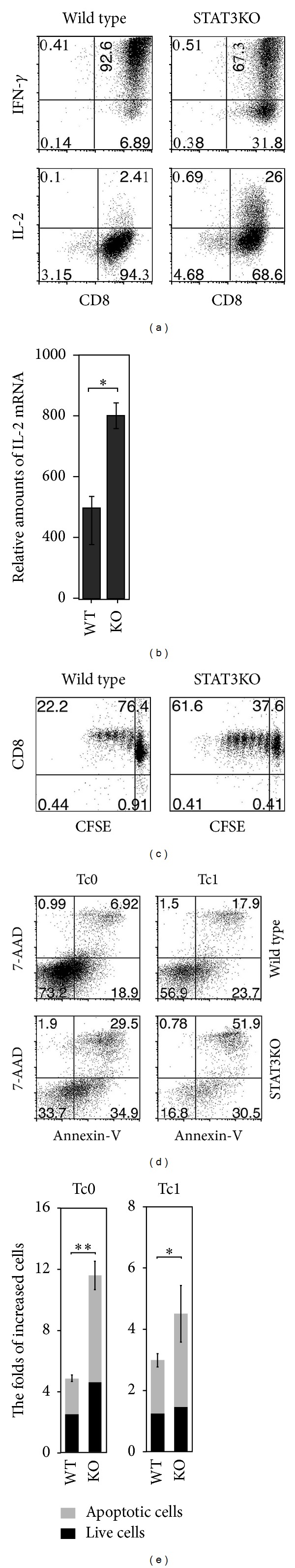
STAT3 inhibits CD8^+^ T cell proliferation and protects T cells from apoptosis. (a, b) WT or STAT3KO naïve T cells were stimulated with anti-CD3/CD28 Abs under nonpolarizing condition for 4 days, and IL-2 or IFN-*γ* expression was detected and quantified by the intracellular cytokine staining assay (a) or RT-qPCR (b). (c) WT or STAT3KO T cells were stimulated *in vitro* with anti-CD3/CD28 Abs, and CFSE dilution analysis was performed after 4 d stimulations. (d) Naïve CD8^+^ T cells were cultured under Tc0 or Tc1 condition for 4 days and stained with Annexin-V and 7-AAD. The cells undergoing apoptosis or necrosis were detected by FACS and cell counting using Vi-Cell XR cell-viability analyzer. (e) The relative percentages of apoptotic and viable cells were derived from the data shown in Figure 2(d) and statistically analyzed with Student's *t*-test. Results are representative of 3 independent experiments.

Previous studies have suggested complex roles of STAT3 in mechanisms that regulate T cell proliferation [9] and that STAT3 and IL-2 signals exert mutually antagonistic effects on CD4^+^ T cell proliferation and Th17 differentiation [23–26]. In this study, we have investigated whether STAT3 plays a role in regulating IL-2 production and proliferation of CD8^+^ T cells. We show here that STAT3-deficient CD8^+^ T cells activated by stimulation with anti-CD3/anti-CD28 Abs produced more IL-2 than WT CD8^+^ T cells as shown by intracellular cytokine staining analysis and RT-qPCR (Figures 2(a) and 2(b)). Results of CFSE dilution assay show that the CD8-STAT3KO T cells divided much faster than WT controls in response to TCR stimulation (Figure 2(c)). This suggests that the loss of STAT3 signaling might have contributed to the observed enhancement of IL-2 production and cellular proliferation by the STAT3-deficient CD8^+^ T cells. Surprisingly, intracellular cytokine staining analysis showed that the frequency of IFN-*γ*-producing cells was substantially decreased (67.3%) in the STAT3-deficient CD8^+^ T cells compared to 92.6% of the WT CD8^+^ T cells WT (Figure 2(a); top panels). In contrast, loss of STAT3 in CD4^+^ T cells impaired Th17 development resulting in concomitant expansion of IFN-*γ*-producing Th1 cells [7]. Thus, data provided here suggests that IFN-*γ* production by activated CD8^+^ STAT3KO T cells is compromised.

T cells with high proliferative capacity produce high levels of IL-2 and are highly susceptible to IL-2-induced AICD [13]. We therefore examined whether the increase of IL-2 production renders the STAT3-deficient CD8^+^ T cells more susceptible to AICD. We stimulated WT or STAT3-deficient CD8^+^ naïve T cells with anti-CD3/CD28 Abs for 4 days under Tc0 or Tc1 polarization condition and determined the number of T cells undergoing apoptosis by the Annexin-V staining assay and FACS. Compared to WT Tc0 and Tc1 cells, higher percentages of the STAT3-deficient CD8^+^ Tc0 or Tc1 cells were undergoing apoptosis after 4 days of TCR activation as revealed by Annexin-V staining (Figures 2(d) and 2(e)). It is however important to emphasize that although similar numbers of cells were used at the beginning of the study, after culturing the cells for 4 days under Tc0 or Tc1 condition, the Tc1 cells experienced more apoptosis. Thus, the total numbers of viable cells acquired were not exactly the same for each group. In addition, the STAT3-deficient CD8^+^ Tc0 or Tc1 cells experienced higher necrotic cell death as indicated by higher percentages of 7-AAD-positive cells (Figures 2(d) and 2(e)). Nonetheless, these results suggest that STAT3 may inhibit IL-2-induced AICD and enhance the survival of CD8^+^ T cells by constraining IL-2 production. 

### 3.3. STAT3 Regulates Proapoptotic and Cell Cycle Regulatory Genes of STAT3KO-CD8^+^ T Cells

**Figure 3:**
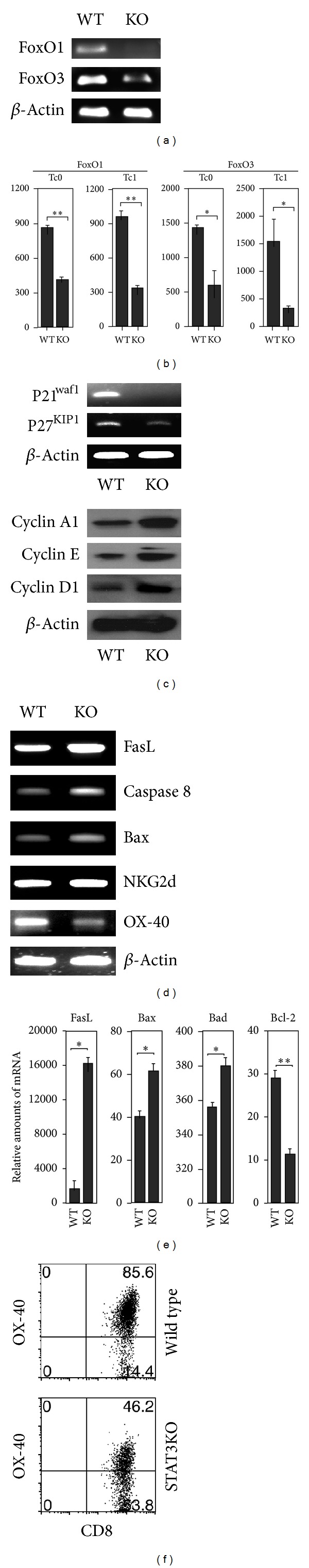
Enhanced proliferation and sensitivity to AICD of STAT3-deficient CD8^+^ T cells correlated with alterations in the expression of proapoptotic, antiapoptosis, and cell-cycle regulatory genes. Naïve CD8^+^ T cells were cultured for 4 days under Tc0 and Tc1 polarization condition. RNA was isolated and analyzed by RT-PCR (a, c, d) or qRT-PCR (b, e). (f) Naïve CD8^+^ T cells were cultured for 4 days under non-polarizing condition, and expression of OX-40 was detected by FACS. Numbers in quadrants represent percentage of CD8^+^ T cells expressing OX-40. Data represent at least 3 independent experiments.

STAT3 regulates proliferation and IL-2 production by CD4^+^ T cells by increasing the expression of transcription factors and cell cycle regulatory genes that maintain T cells in the quiescent state [14, 22]. We examined whether STAT3 also regulates the proliferation and survival of CD8^+^ T cells by upregulating transcription of genes that code for lymphocyte quiescence factors and/or cell cycle regulatory proteins. We isolated naïve CD8^+^ T cells from the lymph nodes of WT or CD4-STAT3KO mice and stimulated the cells with anti-CD3/CD28 Abs for 4 days under Tc0 or Tc1 polarization condition. RNA was isolated from the cells, and RT-PCR or RT-qPCR analysis revealed marked decrease of the transcription of *foxO1* and *foxO3a *genes in the STAT3-deficient CD8^+^ T cells compared to WT CD8^+^ T cells (Figures 3(a) and 3(b)), consistent with the involvement of these class O forkhead transcription factors in regulating lymphocyte proliferation and quiescence [10]. The inverse correlation between the reduction in the levels of FoxO1/FoxO3a mRNA transcripts and enhanced proliferation of STAT3-deficient CD8^+^ T cells supports the notion that STAT3 regulates lymphocyte proliferation through modulation of FoxO gene expression [14]. p21^WAF1^ and p27^Kip1^ are cyclin-dependent kinase inhibitors that regulate transit of proliferating T cells through the G1 stage of the cell cycle, and these cell cycle regulators are direct targets of FoxO proteins [27]. We show here that the transcription of p21^WAF1^  or p27^Kip1^  is also substantially reduced in STAT3-deficient CD8^+^ T cells (Figure 3(c)), and this was accompanied by a corresponding increase in expression of cyclin genes (Figure 3(c)). In addition, transcription of proapoptotic genes (FasL, caspase 8, Bax, and Bad) was elevated in the STAT3-deficient CD8^+^ Tc0 cells (Figures 3(d), and 3(e)), while expression of antiapoptotic genes OX40 and Bcl-2 was decreased (Figures 3(d), 3(e), and 3(f)), consistent with enhanced apoptosis observed in STAT3-deficient CD8^+^ T cells (Figures 2(d), and 2(e)). 

### 3.4. STAT3 Enhances CD8^+^ T Cell Effector Response to HSV-1

**Figure 4:**
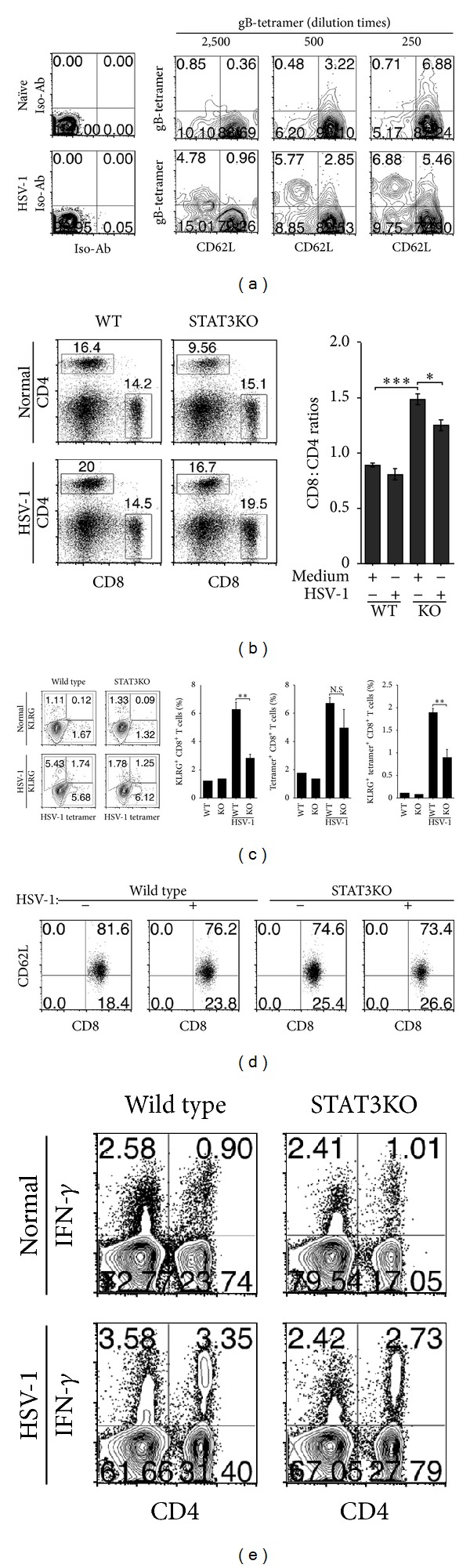
STAT3KO mice are defective in generating HSV-1 specific CD8^+^ effector T cells in response to HSV-1 infection. (a–d) C57BL/6 mice were infected with HSV-1, and immune response to the virus was assessed by FACS analysis. (a) Virus-specific response was established and assessed in the WT mouse by FACS using gB-tetramers. (b–d), Analysis of CD4^+^ and CD8^+^ T cell responses to HSV-1 (b), activated virus-specific CD8^+^ T cells (c), and IFN-*γ* production by the HSV-1-specific CD4/CD8 T cells (d). Cells were gated on CD8 (a, c) or CD3 (b, d), and numbers in quadrants represent percentage of tetramer positive CD8^+^ T cells or T cells expressing KLRG, CD62L, CD4, CD8, or IFN-*γ*. Data represent at least 3 independent experiments.

Host immunity against viruses is mainly mediated by CD8^+^ T cells. It was therefore of interest to investigate whether STAT3 signaling is required for this essential function of cytotoxic T cells. We infected age-matched WT and CD4-STAT3KO mice with the HSV-1 virus strain RE-pICP0-EGFP [11] by intraperitoneal injection. On day 9 after the infection, freshly isolated cells from the spleen were analyzed for the expansion of HSV-1-specific CD8 response using an anti HSV-1 gB tetramer [28]. Dose-dependent titration of the tetramer established a dilution of 1 : 2500 as the optimal tetramer concentration of the tetramer for detection of HSV-1-specific CD8^+^ T cells (Figure 4(a)), and this concentration was used in all subsequent analyses. KLRG-1 (killer cell lectin-like receptor subfamily G member 1) is a transmembrane protein preferentially expressed in natural killer (NK) cells. It is expressed following detection of virus epitopes in context of MHC class I molecules and is therefore a marker of virus-specific CD8^+^ T cell response. Although KLRG is predominantly expressed on NK cells, its expression on CD8^+^ T cells correlates with chronic viral infections. Thus, increased expression of KLRG1 by virus-specific CD8 T cells occurs mainly during persistent antigen stimulation [29]. Interestingly, we observed substantial increases in CD8^+^ T cells in the infected CD4-STAT3KO mice as indicated in the CD8/CD4 ratio (Figure 4(b)). Previous reports have shown that the loss of STAT3 in CD4 compartment caused enhanced proliferation of both CD4^+^ and CD8^+^ T cells [14]. A consequence of the increased proliferative capacity is the enhanced susceptibility to antigen-induced cell death as indicated in this study and previous reports [7, 13]. It is notable that the CD4^+^ T cells appear to be more susceptible to apoptosis as reflected by the relatively higher levels of CD8 : CD4 ratios (Figure 4(b)). Despite the increased frequency of CD8^+^ T cells, CD4-STAT3KO mice contained significantly lower amounts of activated KLRG-1^+^ HSV-1-responsive CD8^+^ T cells (Figure 4(c)). Analysis of CD62L in the CD8^+^ cells after HSV-1 infection thus suggests that the KO mice could mount an efficient primary response while producing less memory cells (Figure 4(d)). In addition, the STAT3-deficient CD8^+^ T cells produced much reduced levels of the anti-inflammatory cytokine IFN-*γ* (Figure 4(e)). It is notable that a similar frequency of gB-tetramer positive CD8 T cells are responding, but the virus-infected WT CD8^+^ T cells express 2.366-fold more KRLG-1 compared to the STAT3-deficient CD8^+^ T cells, suggesting that the knockout cells are responding, but may be mostly SLEC (short lived effector CD8 cells). These results suggest that activation of STAT3 pathways may be required for the development of a functionally fit CD8 effector T cell capable of mounting effective immune responses against HSV-1.

### 3.5. STAT3 Inhibits IL-10-Producing Regulatory CD8^+^ T Cells during Autoimmune Uveitis

**Figure 5:**
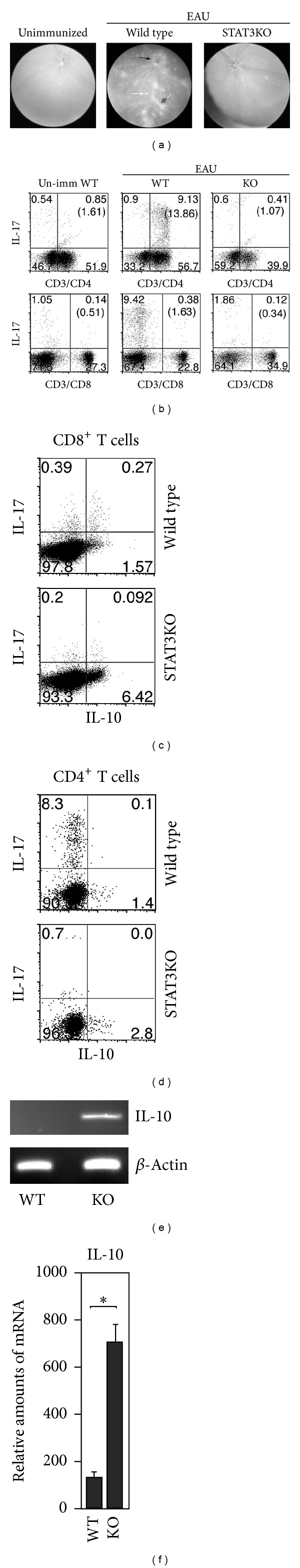
STAT3KO mice induce expansion of IL-10-producing regulatory CD8^+^ T cells during EAU. (a–d) WT or STAT3KO mice were immunized with IRBP in CFA. (a) Fundus images taken on day-21 after immunization. (b) and 21 days after immunization. Freshly isolated LN cells (21 days after immunization) were analyzed by the intracellular cytokine staining (b, c). CD3^+^ T cells were gated, and numbers in quadrants indicate percentage of CD4^+^ or CD8^+^ T cells expressing IL-17A or IL-10. (d, e) CD8^+^ T cells were isolated from the LN by magnetic sorting, and RNA was analyzed for IL-10 expression by RT-qPCR (d) or RT-qPCR (e). Data represent at least 3 independent experiments.

CD4-STAT3KO mice do not develop EAU or EAE [7, 12]. To examine whether functional defects in STAT3KO CD8^+^ T cells contribute to the resistance to developing experimental autoimmune uveitis (EAU), we immunized WT or CD4-STAT3KO mice with IRBP in CFA as described in the methods section. At various time points after immunization, the eyes were examined by fundoscopy, and fundus images taken 21 days after induction of EAU show the development of ocular pathology in the retina of the WT but not the CD4-STAT3KO mice (Figure 5(a)). On day 21 after immunization, we also isolated PBMC and analyzed CD4^+^ and CD8^+^ T cell responses by intracellular cytokine staining assay. We focused on IL-17-expressing T cells because Th17 cells are implicated in the induction and exacerbation of EAU and human uveitis [7, 30]. We observed a significant increase of IL-17-expressing CD4^+^ in WT (13.86%) compared to the paucity of Th17 cells in CD4-STAT3KO (1.07%), and this is consistent with the role of Th17 cells in mediating EAU (Figure 5(a)) [7, 30]. In contrast, WT or STAT3-deficient CD8^+^ T cells comprised 1.63% and 0.34% of the IL-17-producing Tc17 cells, respectively (Figure 5(b)), suggesting that CD8^+^ T cells may not be involved in mediating the disease. It is however of note that IL-10-producing CD8^+^ T cells have recently been shown to play an essential role in the recovery from EAE [31], another CNS autoimmune disease that shares essential attributes of the immune-pathogenic mechanisms with EAU [32]. We therefore isolated cells from the spleen and lymph nodes of WT or CD4-STAT3KO mice 21 days after immunization with IRBP and examined whether the loss of STAT3 affected the generation of this important regulatory CD8^+^ T cell population during EAU. We show here for the first time that the IL-10-producing regulatory CD8^+^ T cell population is indeed recruited into peripheral lymphoid tissues of mice during EAU (Figure 5(c)). Surprisingly, we observed significant expansion of the IL-10-producing CD8^+^ T cell population in these tissues of the IRBP-immunized CD4-STAT3KO mice, with levels more than 4-fold higher compared to WT mice (Figure 5(c)). We also observed a modest increase in IL-10-expressing STAT3-deficient CD4^+^ T cells (Figure 5(d)). CD8^+^ T cells were isolated from the spleen and lymph nodes of day-21 immunized WT or CD4-STAT3KO mice, purified by sorting on magnetic bead, and RNA from the cells was analyzed for IL-10 expression. Result of RT-PCR (Figure 5(e)) or RT-qPCR (Figure 5(f)) analyses confirmed that the IRBP-immunized CD4-STAT3KO mice contained significant levels of CD8^+^ T cells expressing IL-10 mRNA compared to CD8^+^ T cells from the WT mice with EAU (Figures 5(e) and 5(f)). These results uncover a previously unrecognized role of STAT3 in CD8^+^ T cells and suggest that in addition to promoting Th17 disease-inducing activities, STAT3 activation in CD8^+^ T cells may further undermine the recovery from EAU by inhibiting the development or expansion of IL-10-producing CD8^+^ regulatory cells. 

## 4. Discussion

In this study, we have characterized the role of STAT3 pathways in CD8^+^ T cell. Specifically, we examined whether the loss of STAT3 affected the development of the CD8^+^ T cell phenotype or its regulatory functions during HSV-1 infection and mouse uveitis. STAT3-deficient CD8^+^ T cells were found to exhibit an activation phenotype consistent with previous findings in STAT3-deficient CD4^+^ T cells [14, 22]. We also show here that the STAT3-deficient CD8^+^ T cells divided faster than WT CD8^+^ T cells in response to TCR stimulation and their enhanced cellular proliferation was accompanied by marked increase in IL-2 production. It is however notable that the increased production of IL-2 and high proliferative capacity rendered the STAT3-deficient CD8^+^ T cells highly susceptible to AICD.

Similar to STAT3-deficient CD4^+^ T cells, the enhanced proliferation of the STAT3-deficient CD8^+^ T cells derived, in part, from the downregulation of FoxO1, FoxO3A, p21^waf1^, and p27^KIP1^. Our data further suggest that the increased sensitivity to AICD can be attributed to reduced expression of the antiapoptotic proteins, Bcl-2 and OX-40, and up-regulation of the transcription of proapoptotic genes, FasL, Bax, and Bad. Although STAT3 pathways appear to have similar regulatory effects on CD4^+^ and CD8^+^ T cells, the CD8 phenotype appears to be more sensitive to growth inhibitory effects of STAT3. Despite the increased cell death from AICD, we observed significantly higher total numbers of STAT3-deficient Tc0 and Tc1 cells compared to the WT, Tc0, or Tc1. During HSV-1 infection, the overall immune response to the virus was similar between WT and CD4-STAT3KO mice. However, the CD4-STAT3KO mice contained significantly lower amounts of activated KLRG-1^+^ HSV-1-responsive CD8^+^ T cells, underscoring the important role played by STAT3 pathways in promoting CD8^+^ T cell effector responses to virus. 

The production of IFN-*γ* is a hallmark feature of CD8^+^ T cells. Surprisingly, the production of IFN-*γ* was markedly decreased in the STAT3-deficient CD8^+^ T cells (Figures 2(a) and 4(d)), suggesting that STAT3 may promote the expansion of IFN-*γ*-producing Tc1 cells *in vivo*. This contrasts with results from previous studies indicating that CD4-STAT3KO mice exhibit exaggerated increase of IFN-*γ*-producing Th1 cells [7] and argues for diametrically opposite effects of STAT3 on IFN-*γ* expression by CD4^+^ and CD8^+^ T cells. Thus, activation of STAT3 pathways in CD4^+^ T cells may promote Th17 development and inhibit IFN-*γ* production by antagonizing Th1 differentiation and expansion. On the other hand, STAT3 pathways seem to enhance IFN-*γ* production in CD8^+^ T cells by a yet unknown mechanism. Confirmation that STAT3 does indeed transactivate *IFN-*γ** in CD8^+^ T cells will require demonstration by chromatin immunoprecipitation (CHIP) assay that STAT3 binds directly to the *IFN-*γ** promoter or locus. Nonetheless, the involvement of STAT3 in regulating IFN-*γ* production in CD8^+^ T cells is supported by our *in vivo* data showing a 56% increase in IFN-*γ*-expressing CD8^+^ T cells in WT mice infected with HSV-1, while the frequency of IFN-*γ*-producing CD8^+^ T cells in the infected CD4-STAT3KO mice was much reduced (Figure 4(d)).

Another instance where the effect of STAT3 appears to differ between CD4^+^ and CD8^+^ T cells is during the recovery phase of EAU. Although there is strong consensus that EAU and EAE are predominantly CD4^+^ T cell mediated diseases, the role of CD8^+^ T cells has not been clear. However, transfer of IL-10-producing CD8^+^ T cells into mice with EAE dramatically diminished EAE symptoms, indicating that CD8^+^ T cells play a regulatory role in the recovery from EAE [31]. In this study, we detected IL-10-producing CD8^+^ T cells in the spleen and lymph nodes of WT EAU mice (Figure 5(c)). However, in the STAT3KO mice that are resistant to EAU, we observed a dramatic expansion of the IL-10-producing CD8^+^ regulatory T cells compared to WT (1.57% versus 6.42%). This is the first report of the involvement of IL-10-producing regulatory CD8^+^ T cells in EAU and provides confirmation of a previous report in EAE [31].

 Use of the CD4-Cre mouse strain in generating T cell-specific conditional knockout mice has provided a wealth of knowledge on the physiological roles of several transcription factors in CD4^+^ T cells. Unfortunately, after publication of studies utilizing these valuable mouse strains, there is often little or no incentive to investigate its effects on CD8^+^ T cells despite concomitant deletion of the gene of interest in CD8^+^ T cells. This reluctance derives from the implicit assumption that results gleaned from CD4^+^ T cells would also pertain to CD8^+^ T cells. In this study, we have pointed out several crucial points that the effects of STAT3 differ between CD4^+^ and CD8^+^ T cells. Such differences may provide valuable insights into divergent functions of CD4^+^ and CD8^+^ T cells. For example, most focus on the role of STAT3 in autoinflammatory diseases, such as uveitis and multiple sclerosis, has been on its involvement in the differentiation and expansion of pathogenic Th17 cells. However, we have shown here that IL-10-producing regulatory CD8^+^ T cells are generated during EAU and significantly increased in STAT3-deficient mice, suggesting that STAT3 also functions to suppress this regulatory CD8^+^ T cell population that plays a role in the recovery from organ-specific autoimmune diseases. In this context, we posit that the involvement of STAT3 activation in pathogenic mechanisms of autoimmune and infectious diseases may not be confined to CD4^+^ T cells. While STAT3-mediated expansion of Th17 cells may initiate the acute uveitis, aberrant activation of STAT3 by IL-6 during the inflammatory process may promote development of chronic uveitis by inhibiting expansion of CD8^+^ regulatory T cells and thereby impeding recovery from the acute disease. Thus, the recent success in ameliorating uveitis by use of small chemical inhibitors of STAT3 [33] cannot be wholly attributed to inhibition of Th17 cells.
